# gga-miR-101-3p Plays a Key Role in *Mycoplasma gallisepticum* (*HS* Strain) Infection of Chicken

**DOI:** 10.3390/ijms161226121

**Published:** 2015-12-02

**Authors:** Jiao Chen, Zaiwei Wang, Dingren Bi, Yue Hou, Yabo Zhao, Jianjun Sun, Xiuli Peng

**Affiliations:** 1 Key Laboratory of Agricultural Animal Genetics, Breeding and Reproduction, Ministry of Education, Huazhong Agricultural University, Wuhan 430070, China; chenjiao123@webmail.hzau.edu.cn (J.C.); wangzaiwei@webmail.hzau.edu.cn (Z.W.); houyue@webmail.hzau.edu.cn (Y.H.); zyb@webmail.hzau.edu.cn (Y.Z.); 2 China National Key Laboratory of Agricultural Microbiology, Huazhong Agricultural University, Wuhan 430070, China; bidingren@mail.hzau.edu.cn; 3 Department of Biological Sciences and Border Biomedical Research Center, University of Texas at El Paso, El Paso, TX 79968, USA

**Keywords:** chicken, *Mycoplasma gallisepticum* (*HS* strain), gga-miR-101-3p, EZH2

## Abstract

*Mycoplasma gallisepticum* (MG), one of the most pathogenic *Mycoplasma*, has caused tremendous economic loss in the poultry industry. Recently, increasing evidence has suggested that micro ribonucleic acids (miRNAs) are involved in microbial pathogenesis. However, little is known about potential roles of miRNAs in MG infection of chicken. In the present study, using miRNA Solexa sequencing we have found that gga-miR-101-3p was up-regulated in the lungs of MG-infected chicken embryos. Moreover, gga-miR-101-3p regulated expression of the host enhancer of zeste homolog 2 (EZH2) through binding to the 3’ un-translated region (3’-UTR) of *EZH2* gene. Over-expression of gga-miR-101-3p significantly inhibited EZH2 expression and hence inhibited proliferation of chicken embryonic fibroblast (DF-1 cells) by blocking the G1-to-S phase transition. Similar results were obtained in MG-infected chicken embryos and DF-1 cells, where gga-miR-101-3p was significantly up-regulated, while EZH2 was significantly down-regulated. This study reveals that gga-miR-101-3p plays an important role in MG infection through regulation of EZH2 expression and provides a new insight into the mechanisms of MG pathogenesis.

## 1. Introduction

*Mycoplasmas* are cell wall-less prokaryotes that are widespread in nature either as parasites or as commensals in eukaryotic hosts. Mycoplasmosis, the diseases caused by *Mycoplasmas* occur in animals and humans and have multiple clinical appearances [1,2,3,4,5,6,7]. As one of the most important *Mycoplasma* species [8], MG causes for avian chronic respiratory diseases, especially in chickens and turkeys in the world [9,10,11], with featured inflammation in the trachea, air sacs and lungs [12]. MG is known to invade, survive and multiply inside a variety of non-phagocytic cells, such as chicken fibroblasts and HeLa cells [13,14,15,16]. MG-HS strain is a virulence strain isolated from the chicken farms in Hubei Province of China [17,18]. MG infection in chicken farms usually lasts for a long time and is very difficult to be eliminated completely. Infected birds become life-long carriers through horizontal and vertical transmission [19]. While there are vaccines (e.g., F-strain, ts-11-strain, and 6/85-strainvaccines) and antibiotics (e.g., Tylosin) available for prevention and treatment of MG infection, antibiotics and vaccines have no impact on the life-long carrier status of infected poultry. The increased MG epidemic has caused great economic losses in the poultry industry worldwide [20].

MiRNAs are a class of small, non-coding, single-stranded RNAs consisting of 22–25 nucleotides. Since the discovery of the first miRNA, lin-4, in *Caenorhabditis elegans* two decades ago [21], over 24,000 curated miRNA entries have been identified from various species [22] For chicken, more than 859 miRNAs have been identified so far [22], and only a few of them have been studied. It is well known that miRNAs can negatively regulate gene expression at the post-transcriptional level via an RNA interference (RNAi) mechanism [23]. Partial or full complementary pairing of miRNAs with target mRNAs in the 3’-UTR causes translational repression and/or degradation of mRNAs, which result in silencing of the target gene [24,25,26,27,28]. Therefore, down-regulation of miRNAs usually increases expression of targeted gene(s), whereas up-regulation of miRNAs leads to suppression of target genes. MiRNAs offer a fast, energy-saving and fine-tuning mechanism for post-transcriptional control of protein production [27]. It is believed that up to 30% of human protein coding genes may be regulated by miRNAs [29].

Current studies have suggested that miRNAs play important roles in various physiological and pathological processes in the avian world [23,30]. For instance, miRNAs are involved in poultry diseases, such as avian leucosis [31,32], avian influenza [33], infection bursal disease [34], Marek’s disease [35,36,37,38], and ovarian carcinoma [39].

A number of studies have indicated that miR-101 is involved in a variety of diseases [40,41,42,43,44]. Recently, we investigated the miRNA expression profiling in the MG-infected lungs *vs.* the non-infected lungs of specific-pathogen-free (SPF) chicken embryos by Solexa deep sequencing (lab unpublished data). The preliminary data showed that gga-miR-101-3P was up-regulated in the MG-infected lungs, compared with non-infected lungs, suggesting that gga-miR-101-3P may play an important role in MG infection of chicken. In the present study, we identified EZH2 as the target of gga-miR-101-3P and tested the effects of the miRNA on expression of EZH2 and cell growth in the context of MG infection.

## 2. Results

### 2.1. Prediction of the Target Gene of gga-miR-101-3p

**Figure 1:**
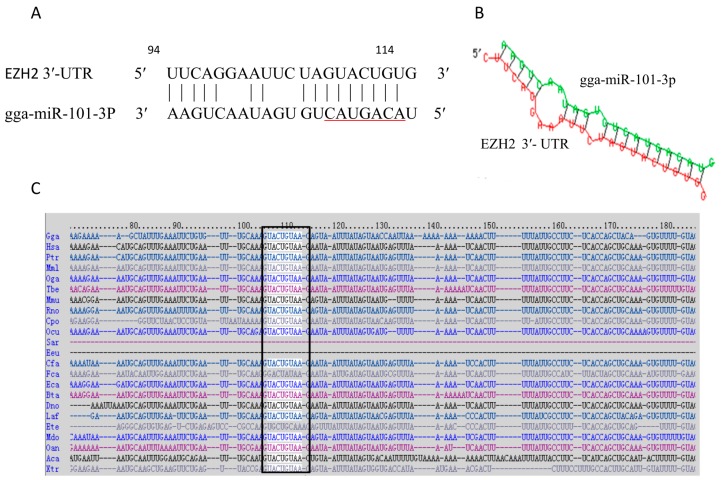
Prediction of the target gene of gga-miR-101-3p. (**A**) Sequence alignments of gga-miR-101-3p and the target site in the 3′-UTR of EZH2. The seed sequence of gga-miR-101-3p is underlined, and the complementary nucleotides between gga-miR-101-3p and EZH2 3′-UTR are indicated; (**B**) Secondary structure of the RNA duplex of gga-miR-101-3p and EZH2 3′-UTR target site (Red: Target sequence; Green: gga-miR101-3p); (**C**) Sequence alignment of EZH*2* 3′-UTR from different species. The conserved target sequences are highlighted. Species abbreviations refer to the website link [56].

In our previous studies, we found that gga-miR-101-3p was up-regulated in the lungs of the infected chicken embryos by miRNA solexa sequencing (lab unpublished data). Since it is well documented that miRNAs exert their function through regulating expression of their target gene(s) [45], we sought to identify the direct target of gga-miR-101-3p involved in MG-HS infection. The prediction software/servers from TargetScan [46], miRBase [22], miRecords [47], and miRDB [48] were used to search the putative protein-coding gene targets of gga-miR-101-3p. Data collection and analysis revealed that EZH2 was as a potential target gene of gga-miR-101-3p (score 98.36). The predicted target site is at 94–114, with the seed region of miR-101 at 107-113 (Figure 1A). Then, we used RNAhybrid [49] to analyze the duplex and the minimum free energy (mFE between gga-miR-101-3p and EZH2 3’-UTR. MFE of RNA duplex is about −23.8 kCal/mole, indicating it has a high stability (Figure 1B). The putative target site on the 3’-UTR of EZH2 is highly conserved in a wide range of species, including human, mouse, dog, monkey and platypus (Figure 1C). We used AmiGo [50] to analyze EZH2 functions in *Gallus gallus*. EZH2 can positively regulate mitogen-activated protein kinase (MAPK) activity and cell proliferation.

Upon recognition of pathogen associated molecular patterns (PAMPs), MAPKs are activated rapidly, leading to the production of pro-inflammatory cytokines to defend against microbial infection. However, one can imagine that MG-induced up-regulation of gga-miR-101-3p in the lung tissues of chicken embryos may repress the expression of EZH2, which, in turn, prolong the activation of MAPKs, and increase tissue damage in hosts by MG infection. It is also reported that EZH2 plays an important role in development and differentiation of T cells and B cells and in promoting both regulatory and effector responses [51,52,53,54,55]. Thus, we hypothesize that gga-miR-101-3p is involved in MG-infection through regulation of EZH2 expression.

### 2.2. EZH2 Is the Direct Target of gga-miR-101-3p

**Figure 2:**
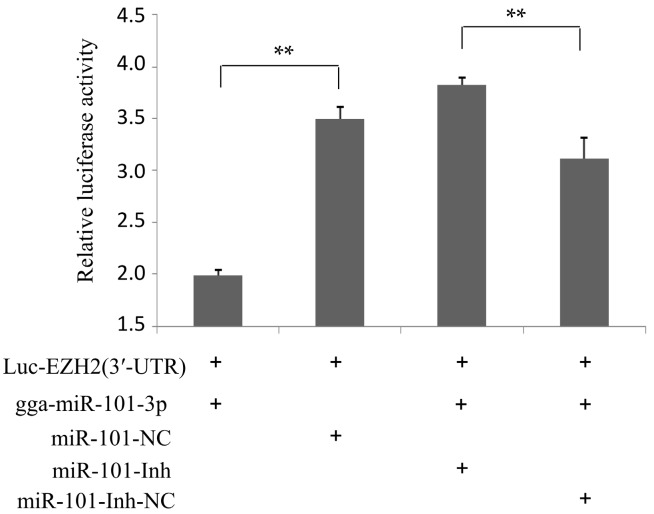
EZH2 is the direct target of gga-miR-101-3p. DF-1 cells were co-transfected with Luc-EZH2 (3′-UTR) and the indicated RNA oligonucleotides. At 24 h of post-transfection, the cells were assayed for both firefly and renilla luciferase using the dual-luciferase glow assay. Three independent experiments were performed and each sample was tested in triplicates. Data are expressed as means ± SD. Two-tailed Student’s *t*-test was used to analyze the significant differences (** *p* < 0.01).

To further verify whether gga-miR-101-3p directly targets to the 3′-UTR of EZH2 in DF-1 cells, we performed a luciferase reporter assay. The target sequence of EZH2 3′-UTR was cloned into a luciferase reporter vector to generate Luc-EZH2 (3′-UTR). Expression of luciferase can be inhibited by binding of gga-miR-101-3p to 3′-UTR of EZH2. Co-transfection of Luc-EZH2 (3′-UTR) with gga-miR-101-3p mimics (gga-miR-101-3p) in DF-1 cells resulted in a significant decrease of luciferase activity, while transfection of a non-specific RNA (miR-101-NC), did not affect luciferase activity (Figure 2). When the gga-miR-101-3p specific inhibitor (miR-101-Inh) was transfected into the DF-1 cells, the luciferase activity was significantly increased even in the presence of gga-miR-101-3p. As expected, a negative control for the gga-miR-101-3p specific inhibitor (miR-101-Inh-NC) did not inhibit EZH2 3′-UTR luciferase activity (Figure 2), which demonstrated that gga-miR-101-3p negatively regulated expression of EZH2 by binding to the complementary sequence in the 3′-UTR of EZH2 in a direct and sequence-specific manner.

### 2.3. Effects of gga-miR-101-3p on EZH2 Expression

**Figure 3:**
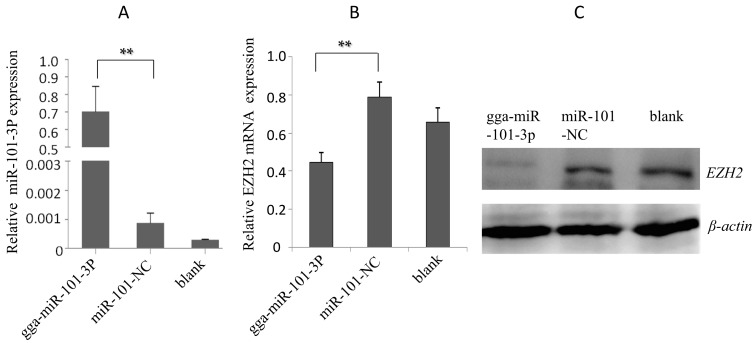
Over-expression of gga-miR-101-3p repressed EZH2 expression. (**A**) Over-expression of gga-miR-101-3p in DF-1 cells; (**B**) The mRNA level of EZH2 in DF-1 cells overexpressing gga-miR-101-3p was reduced. Error bars represent the mean ± SD of triplicate experiments; (**C**) Western blot analysis of EZH2 protein expression in DF-1 cells transfected with gga-miR-101-3p. A mock transfection was set as a blank; the expression of β-actin was used as a loading control. Each experiment was repeated three times, and each sample was assayed in triplicate. The asterisks represented statistically significant differences (** *p* < 0.01).

**Figure 4:**
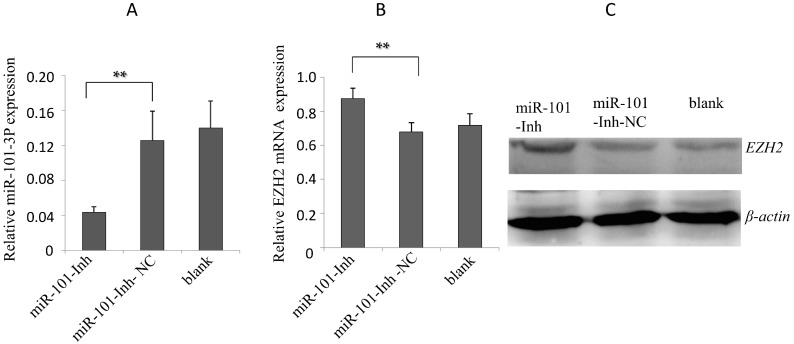
Inhibition of gga-miR-101-3p increased EZH2 expression. (**A**) Transfection of miR-101-Inh reduced the expression of gga-miR-101-3p in DF-1 cells, as detected by RT-qPCR; (**B**) Transfection of miR-101-Inh increased the mRNA expression of EZH2 in DF-1 cells. Error bars represent the mean ± SD of triplicate experiments; (**C**) Transfection of miR-101-Inh increased protein expression of EZH2 in DF-1 cells. A mock transfection was set as a blank, and the expression of β-actin was used as a loading control. Each experiment was repeated three times, and each sample was assayed in triplicate. The asterisks represented statistically significant differences (** *p* < 0.01).

To determine the roles of gga-miR-101-3p in regulation of EZH2 expression, we tested effects of over-expression or suppression of gga-miR-101-3p on EZH2 expression in DF-1 cells. Through transient transfection, gga-miR-101-3p was over-expressed in DF-1 cells (Figure 3A). As a negative control, miR-101-NC was over-expressed in DF-1 cells through transient transfection. In addition, a mock transfection was set as blank. Over-expression of gga-miR-101-3p, but not miR-101-NC, resulted in a significant decrease of EZH2 expression at both mRNA and protein levels (Figure 3B,C). Subsequently, over-expression of miR-101-Inh drastically inhibited gga-miR-101-3p expression (Figure 4A), which, in turn, increased EZH2 expression at both mRNA and protein levels (Figure 4B,C). The data suggest that gga-miR-101-3p negatively regulates expression of EZH2 expression in DF-1 cells through binding to the 3′-UTR of EZH2.

### 2.4. Effects of gga-miR-101-3P on Cell Proliferation and Cell Cycle

**Figure 5:**
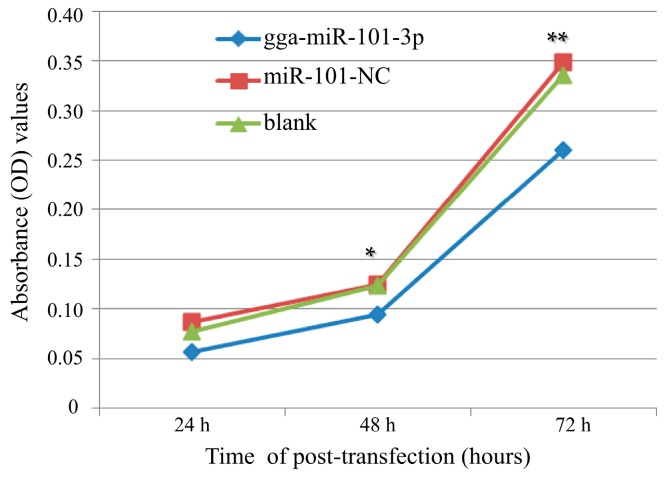
Over-expression of gga-miR-101-3p inhibited DF-1 cell proliferation. DF-1 cells were transfected with gga-miR-101-3p or the negative control. A mock transfection was set as a blank. At 24, 48, 72 h of post-transfection, the cell proliferation was measured using an MTT-Cell Proliferation and Cytotoxicity Assay Kit (Beyotime, Beijing, China). Plotted means and standard deviation were computed from data of three independent experiments, and the relative cell growth is shown. The asterisks represented statistically significant differences (* *p* < 0.05, ** *p* < 0.01).

To further explore the role of gga-miR-101-3p in MG-HS infection, we examined the effects of gga-miR-101-3p on DF-1 cell proliferation. DF-1 cells were transfected with gga-miR-101-3p or miR-101-NC, and then cultured for various periods of time (24 h, 48 h and 72 h). In addition, a mock transfection was set as a blank. Cell proliferation assay showed that over-expression of gga-miR-101-3p significantly reduced the proliferation of DF-1 cells at 48 h and 72 h of post-transfection, compared to miR-101-NC or the blank control (Figure 5).

**Figure 6:**
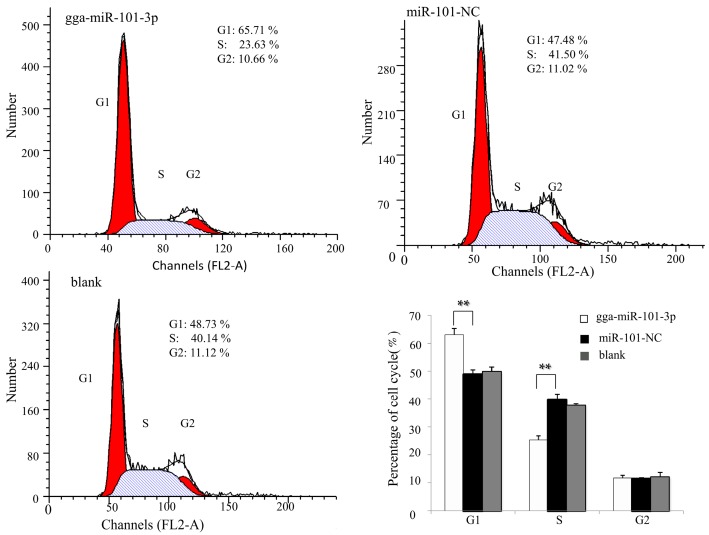
Over-expression of gga-miR-101-3p arrested DF1 cells at G1 phase. The DF-1 cells were transfected with gga-miR-101-3p or the negative control. A mock transfection was also performed as blank control. At 48 h of post-transfection, the cell phase distribution was analyzed by flow cytometry. Three independent experiments were performed in triplicate. Data are presented as means ± SD. Two-tailed Student’s *t*-test was used to analyze significant differences (** *p* < 0.01).

Next, we assessed the effects of gga-miR-101-3p on cell cycle distribution by flow cytometry. In DF-1 cells, gga-miR-101-3p significantly reduced the percentage of S-phase cells, but increased the percentage of G1-phase cells (Figure 6). Taken together, these results indicate that over-expression of gga-miR-101-3p induces G1-phase arrest, which results in inhibition of cell growth and proliferation.

### 2.5. Expression of gga-miR-101-3p and EZH2 in MG-infected DF-1 Cells and Chicken Embryos

**Figure 7:**
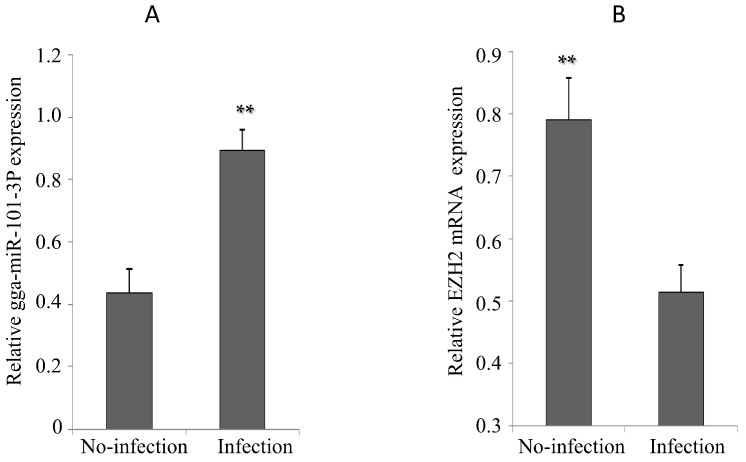
Effects of MG infection on the expression of gga-miR-101-3P and *EZH2* in DF-1 cells. The DF-1 cells were infected by MG as described in section Material and Methods, and the total RNA was extracted. (**A**) gga-miR-101-3p expression was assessed by real-time quantitative reverse transcriptase polymerase chain reaction (RT-qPCR) using 5S-rRNA as an internal quantitative control; (**B**) EZH2 mRNA expression was assessed by RT-qPCR. GAPDH was used as an internal quantitative control. Three independent experiments were performed in triplicate, plotted data points referred to the means ± standard deviations and asterisks represented statistically significant differences (** *p* < 0.01).

**Figure 8:**
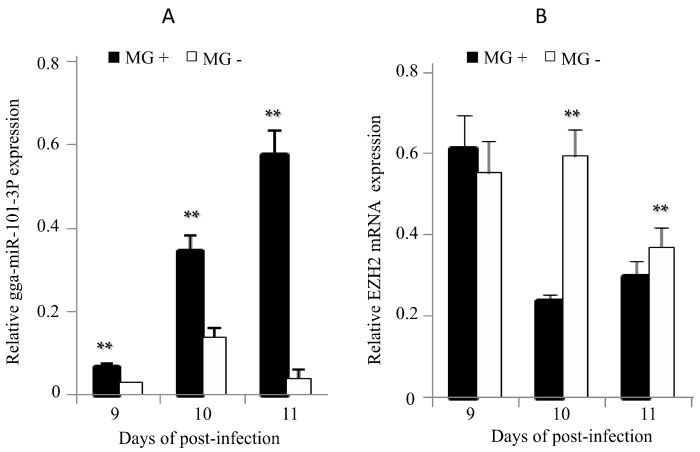
Effects of MG infection on the expression of gga-miR-101-3P and *EZH2* in the lungs of chicken embryos. The chicken embryos were infected by *MG-HS* as described in section Material and Methods. On the 9th, 10th, and 11th days of post-infection, the lungs of the infected chicken embryos were processed and the expression of gga-miR-101-3p (**A**); and EZH2 (**B**) was measured by RT-qPCR. Three independent experiments were performed in triplicate, plotted data points referred to the means ± standard deviations and asterisks represented statistically significant differences (** *p* < 0.01).

Upon MG infection, gga-miR-101-3p expression in DF-1 cells was significantly up-regulated (Figure 7A), while EZH2 expression was significantly down regulated (Figure 7B). The chicken embryos at the 9th day of hatching (total 21 days of egg hatching) were infected by MG-HS. On the 9th–11th days of post-infection (equivalent to the 17th–19th days of egg hatching), we observed that gga-miR-101-3p expression was significantly higher in the lungs of the MG-infected chicken embryos than the non-infected group (Figure 8A). As expected, EZH2 expression showed opposite patterns as gga-miR-101-3p on the 10th–11th days of post-infection (equivalent to the 18th and 19th days of egg hatching) (Figure 8B).

## 3. Discussion

It is well documented that miRNAs can bind and silence multiple target genes, which regulate gene expression and affects malignant cellular behaviors [57,58]. Host miRNAs regulate expression of genes from pathogenic microorganism, and pathogenic microorganisms develop highly sophisticated mechanisms to evade host immune responses. In chronic respiratory diseases of chickens, some host miRNAs are aberrantly expressed. Thus, it is important to understand the roles and underlying mechanisms by which miRNAs affect *Mycoplasma* infection.

Our preliminary study showed that gga-miR-101-3p was up-regulated in MG-infected SPF chicken embryos (lab unpublished data). Consistent to our previous study, we found here that gga-miR-101-3p is up-regulated in MG-infected DF-1 cells and in the lung tissues of MG-infected chicken embryos. More importantly, we identified EZH2 as the target of gga-miR-101-3P. Gga-miR-101-3p negatively regulates the expression of *EZH2* gene by binding to complementary sequence in the 3′-UTR of EZH2. Analysis by AmiGo software shows that EZH2 functions in *Gallus gallus* and EZH2 can positively regulate MAP kinase activity and cell proliferation [50].

*Mycoplasma* can induce a series of pro-inflammatory cytokines, apoptosis, or necrosis in a variety of cell types [59,60,61]. MAPKs are pivotal mediators of innate immune responses to microbial infection. Upon recognition of PAMPs, MAPKs are activated to produce pro-inflammatory cytokines to defend against microbial infection. In our study, gga-miR-101-3p was up-regulated in MG-infected SPF chicken embryos, which repressed the expression of EZH2. Thus, it is reasonable to believe that MG inhibits activation of MAPKs through up-regulation of gga-miR-101-3p and down-regulation of EZH2. The miRNA family of miR-101 play an important role for in regulating innate immune responses to infection. It has been reported that it inhibits a dual specific phosphatase that deactivates MAPKs [43,62], and it also regulates the MAPK response and affects the secretion of the downstream inflammatory cytokines, such as TNF-α, IFN, IL-1, IL-6 [42].

In the present study, we found that over-expression of gga-miR-101-3p leads to cell cycle arrest. *EZH2* is a growth suppressor gene and involved in the regulation of cell cycle progression and proliferation [63,64].

Recent studies showed that miR-101 could target EZH2 in a variety of cancers. During prostate cancer progression, miR-101 was down-regulated, and over-expression of miR-101 suppressed proliferation of prostate cancer cells [65,66,67]. It is also reported that miR-101 inhibits lung cancer invasion through regulation of EZH2 [41], which inhibits cell proliferation and invasion, but enhances paclitaxel-induced apoptosis in non-small cell lung cancer [40]. Moreover, miR-101 is down-regulated in bladder transitional cell carcinoma, and directly represses EZH2 [68,69]. The miR-101 functions as a tumor suppressor in human retinoblastoma cells by targeting EZH2. It also significantly inhibits cellular proliferation, migration and invasion of gastric cancer cells by targeting EZH2 [70]. Ectopic expression of miR-101 significantly reduced cell growth and proliferation in retinoblastoma through directly targeting EZH2, which was associated with increased G1 phase arrest and cell apoptosis [71]. The miR-101 negatively regulates EZH2 in ovarian cancer cell lines, and miR-101 over-expression resulted in decreased cellular proliferation and migration [72]. Research has shown that miR-101 has been linked to tamoxifen and fulvestrant resistance by its targeting of EZH2 [73]. Both miR-101 and EZH2 are highly conserved in many different species from chicken to human (Figure 1C), suggesting that miR-101-EZH2 is a common pathway in regulating immune response in vertebrates. Thus, it is reasonable to believe that the up-regulation of gga-miR-101-3p in the MG-infected chicken inhibits EZH2 expression, which in turn suppresses proliferation and cell cycle in chicken cells.

EZH2 also plays important roles in the regulation of T cell activity. Recent studies found that both EZH2-deficient regulatory T (Treg) cellsand effector T (Teff) cells were functionally impaired *in vivo*. Treg cells failed to constrain autoimmune colitis, while Teff cells neither provided a protective response to *T. gondii* infection nor mediated autoimmune colitis [74]. The expression of the BMI-1-and EZH2-containing PcG complexes in mature T cells is mutually exclusive, but they both play important roles in various stages of T cell differentiation [75]. EZH2 is highly expressed in developing murine lymphocytes and plays a critical role in early B cell development, rearrangement of the immunoglobulin heavy chain gene and regulation on histone H3 methylation in early B cell progenitors [54].

Here, we observed that, upon MG infection, gga-miR-101-3p is up-regulated in the lung tissue. One can imagine that the up-regulated gga-miR-101-3p would inhibit EZH2 expression, which, in turn, impairs T-cell function. Therefore, gga-miR-101-3p and EZH2 may be a privilege niche for MG to overcome the host immunity. Both gga-miR-101-3p and EZH2 can be used as potential diagnostic biomarkers and therapeutic targets in treatment and prevention of MG infection.

Since both miR-101 and EZH2 are highly conserved in a wide range of species (Figure 1C), the results obtained in chicken in this study will provide useful information and insights for the studies of other species.

## 4. Materials and Methods

### 4.1. Predication of gga-miR-101-3p Target Genes

The databases miRDB [48] and TargetScan [46] were used to search the target genes of gga-miR-101-3p. The duplex and the minimum free energy (mFE) between gga-miR-101-3p and 3′-UTR of the potential targets were analyzed by RNA hybrid [49].The conservatism of the target gene was analyzed by TargetScan [46]. AmiGo [50] was used to analyze functions of the target genes of gga-miR-101-3p in *Gallus gallus.*

### 4.2. Design of DNA Primers and Synthesis of RNA Oligonucleotides

All sequences of the primers used in this study are shown in Table 1. All RNA oligonucleotides was designed and synthesized by GenePharm (Shanghai, China).The gga-miR-101-3p mimics sequences was 5′-GUACAGUACUGUGAUAACUGAA-3′. A non-specific miRNA was used as a negative control for gga-miR-101-3p: sense 5′-UUCUCCGAACGUGUCACGUTT-3′ and antisense 5′-ACGUGACACGUUCGGAGAATT-3′. An miRNA inhibitor sequences were as follows: gga-miR-101-3p inhibitor, 5′-UUCAGUUAUCACAGUACUGUAC-3′; random miRNA inhibitor negative control, 5′-CAGUACUUUUGUGUAGUACAA-3′.

**Table 1:** Sequences of DNA primers.

Name	Primer Sequence (5′–3′)	Accession No.
**Primers for 3′-UTR Cloning**		
EZH2 3′-UTR-F	GCGCTCGAGCTGCCTTATCTTC	XM 004935061
EZH2 3′-UTR-R	ATAGCGGCCGCTAGCTGGTGAGA	XM 004935061
**Primers for RT-qPCR**		
GAPDH-F	GAGGGTAGTGAAGGCTGCTG	NM 204305
GAPDH-R	CACAACACGGTTGCTGTATC	NM 204305
EZH2-F	TGCCTATAATGTACTCATGGTCAC	XM 004935061
EZH2-R	TCATCATTGATAAATCCACACTCT	XM 004935061
RT-gga-miR-101-3p	CTCAACTGGTGTCGTGGAGTCGGCAATTCAGTTGAGTTCAGTTA	MIMAT0001185
gga-miR-101-3p-F	CTGGTAGGGTACAGTACTGTGATA	MIMAT0001185
gga-miR-101-3p-R	CTGGTGTCGTGGAGTCGGC	MIMAT0001185
gga-5s-rRNA-F	CCATACCACCCTGGAAACGC	
gga-5s-rRNA-R	TACTAACCGAGCCCGACCCT	

### 4.3. Cell Culture

DF-1, obtained from American Type Culture Collection, is an immortalized chicken embryonic fibroblast cell line. DF-1 was cultured in Dulbecco’s modified eagle medium (DMEM) (Invitrogen, Carlsbad, CA, USA) supplemented with 10% fetal bovine serum (FBS) (Invitrogen, Carlsbad, CA, USA), 100 U/mL penicillin G and 100 μg/mL streptomycin. Cells cultured in a humidified incubator were grown and maintained at 39 °C with 5% CO_2_.

### 4.4. Plasmid Construction

To construct the dual luciferase reporter plasmid, the full length or the fragments of EZH2 3′-UTR covering the putative gga-miR-101-3P binding site were amplified by RT-PCR from the cDNA extracted from the lung tissues of chicken. The amplified products were sub-cloned into *Xho* I and *Not* I sites of psi-CHECK™-2 vector (Promega, Madison, WI, USA). The primers were listed in Table 1. All constructs were verified by DNA sequencing. In addition, all transfection experiments were performed in triplicate.

### 4.5. Dual-Luciferase Reporter Assay

Dual luciferase reporter assay was comprised of two reporters. One is Renilla luciferase and the other is a firefly luciferase in pmirGLO containing the assayed 3′-UTR sequence. For luciferase reporter assay, DF-1 cells were plated 1 day before transfection. On the 2nd day, 200 ng of luciferase reporter plasmid and 10 pmol of the indicated RNA oligonucleotides were also transfected into DF-1 using Lipofectamine 2000 (Invitrogen Life Technologies, Carlsbad, CA, USA). The cells were collected at 48 h of post-transfection, and the dual-luciferase activity assay was performed according to the manufacturer’s instructions (Promega, Medison, WI, USA). Luciferase activity was detected using a Lumat LB 9507 Ultra-Sensitive Tube Luminometer (Titertek Berthold, Nanjing, China). Firefly luciferase activity of each sample was normalized by Renilla luciferase activity. All transfection experiments were performed in triplicate and repeated at least three times.

### 4.6. Reverse Transcription and Quantitative RT-PCR Analysis

Expression of gga-miR-101-3p and EZH2 was measured using RT-qPCR. The total RNA from the cultured cells and the frozen lung tissue specimens of chicken embryos were isolated using TRIzol Reagent (Invitrogen, Carlsbad, CA, USA) and purified using RNeasy mini columns according to the manufacturer’s instructions (Qiagen; Valencia, CA, USA). Then using the Prime Script™ RT reagent kit with gDNA eraser (TaKaRa, Tokyo, Japan), we performed RT-PCR, in which 1μg of total RNA from each samples was reverse transcribed into cDNA. The Real-time PCR was performed in a TransStart Top Green qPCR SuperMix (TRANSGEN, Beijing, China) on CFX96 or CFX384 Touch™ (Bio-Rad, Hercules, CA, USA). The relative mRNA levels were calculated using 2^−∆∆*C*t^ method [76]. The data were analyzed using 7500 software v.2.0.1 (Applied Biosystems, Foster, CA, USA) with the automatic *C*_t_ setting for adapting baseline and threshold for *C*_t_ determination. 5S-RNA and glyceraldehyde-3-phosphate dehydrogenase (GAPDH) were used as internal controls, respectively. The experiment was repeated three times. The primers were listed in Table 1.

### 4.7. Western Blot Analysis

The DF-1 cells were cultivated in 6-well plates, 10 pmol of the indicated RNA oligonucleotides were transfected into DF-1 using Lipofectamine 2000 (Invitrogen Life Technologies, Carlsbad, CA, USA). The total proteins were isolated at 60 h post-transfection using RIPA-buffer (Beyotime, Beijing, China) supplemented with 100 mM phenylmethanesulfonyl fluoride (PMSF). The total protein extracts were measured by the BCA protein assay reagent kit (Beyotime, Beijing, China), 10 μg of protein were fractionated on 12% SDS-polyacrylamide gel electrophoresis (SDA-PAGE) and transferred by electrophoresis to polyvinylidene fluoride (PVDF) membranes (Beyotime, Beijing, China). Membranes were blocked in 5% (*w*/*v*) fat free milk for 1 h at room temperature. The membrane was incubated overnight with the appropriate primary antibodies, including goat polyclonal anti-EZH2 (Santa Cruz Biotechnology, Inc., Santa Cruz, CA USA) and β-actin, which served as a protein loading control. Then, the membrane was washed and incubated with rabbit anti-goat secondary antibody for 1 h. Protein on the membrane was detected using an ECL™ detection system (Bio-Rad, Hercules, CA, USA) after washing in TBST. All experiments were repeated three times.

### 4.8. Cell Proliferation and Cell Cycle Assay

DF-1 cells were plated at a density of 2 × 10^4^ cells/well in flat-bottom, 96-well cell culture plate and allowed to grow for 4 h at 39 °C with 5% CO_2_. The DF-1 cells were then transfected with the indicated RNA oligonucleotides. At 24 h, 48 h, 72 h of post-transfection, the cells were incubated with 10 μL of 3-(4,5-dimethylthiazol-2-yl)-2,5-diphenyltetrazolium bromide (MTT) (5 mg/mL) for 4 h at 39 °C. 100 μL of Dimethyl Sulphoxide (DMSO) was added to solubilize formanzan. Cell proliferation was measured using a MTT cell proliferation and cytotoxicity assay kit according to the manufacturer’s instructions (Beyotime, Beijing, China). The absorbance at 595 nm of each well was scanned in a microplate reader (Bio-Rad, Hercules, CA, USA).

The cell cycle assay was performed in six 6-well plates. Similarly, DF-1 cells were transfected with the RNA oligonucleotides. At 48 h of post-transfection, cell cycle was analyzed in a flow cytometry using a cell cycle detection kit (KeyGEN, Nanjing, China). At least eight replicate wells were included for each experimental group, and all experiments were repeated independently three times.

### 4.9. Mycoplasma Strains and Growth Condition

MG-HS was isolated from a chicken farm in Hubei province, China [17,18]. MG-HS was deposited and donated by State Key Laboratory of Agricultural Microbiology, College of Veterinary Medicine, Huazhong Agricultural University (Wuhan, China). MG-HS were cultured at 37 °C in modified FM-4 medium supplemented with 12% (*v*/*v*) porcine serum and 10% yeast extract until mid-log phase. The concentration of MG-HS is determined by acid-mediated shift of phenol red dye from red to orange as previously described [17]. The number of viable *Mycoplasmas* in a suspension was then determined by color changing units assay [77].

### 4.10. Infection Experiments

MG infection experiments were divided into experimental group and control group, and carried out in 6-well plates. Cell monolayers were detached from cell culture vials by trypsin treatment, seeded in 6-well plates evenly, and incubated in the medium without antibiotics. The cells of experimental group at 80%–90% confluency were infected with 100 μL of MG at mid exponential phase (1 × 10^10^ CCU/mL). At the 24 h of post-infection, the cells in the two groups were collected with Trizol (Invitrogen, Carlsbad, CA, USA) for further use.

Two-hundred embryos of White Leghorn SPF chicken were used for the MG challenge experiment. At the 9th hatching day, 100 chicken embryos were injected with 300 μL of MG-HS strains at 10^10^ CCU/mL into allantoic cavity. The remaining 100 chicken embryos were injected with the same dosage of diluent as controls. The viability of chick-embryos was examined by eyes under a candling machine. The dead chick-embryos were eliminated. Chicken embryo mortality rate of infection group and control group were 12.3% and 7%, respectively. The whole lung tissue samples from 6 infected live chicken embryos and 6 controls were collected on the 9th–11th days of post-infection, and stored in the RNA fixer (BioTeke Co., Ltd., Beijing, China).

### 4.11. Statistical Analysis

Data are expressed as the mean value ± SD of at least three independent experiments. Statistically significant differences were assessed using Student’s *t*-test. *p*-values < 0.05 were considered significant. Significant differences were denoted as * *p* < 0.05, ** *p* < 0.01.
